# Optimising individual and community involvement in health decision-making in general practice consultations and primary care settings: A way forward

**DOI:** 10.1080/13814788.2020.1861245

**Published:** 2020-12-18

**Authors:** Anne E. MacFarlane

**Affiliations:** School of Medicine and Health Research Institute, University of Limerick, Limerick, Ireland

**Keywords:** General practice, primary care, patient-centred care, shared decision-making, community participation, public and patient Involvement in research

## Abstract

The World Health Organisation Alma-Ata Declaration on Primary Healthcare, and the more recent Astana Declaration from the Global Conference on Primary Healthcare, emphasise the involvement of individuals and communities in health decision-making about their individual health care, service delivery and policy development. Increasingly, health funding agencies and academic publishers like the BMJ require Public and Patient Involvement in health research. These imperatives cover health decision-making about different issues in different settings. In this position paper, I argue that individual and community involvement in health decision-making are core to, and useful for, the discipline of general practice but may not be equally familiar or routinised practices in European primary care settings. I use the social science concept of participatory spaces, to describe three overlapping forms of involvement – shared decision-making (SDM) in clinical care, community participation to develop services and Public and Patient Involvement in research. I refer to evidence of implementation challenges for these forms of involvement and provide insights about how to routinise them with reference to the need for these practices to make more sense to general practitioners, for general practitioners to have more time and resources to incorporate them into their daily work and for more research to understand the power dynamics involved. We need leadership in our discipline, and partnership working with policymakers, patient and community organisations, to progress these issues and enable us to optimise benefits for general practitioners, patients and the broader practice population.

KEY MESSAGES

- Individual and community involvement in health decision-making is useful for general practice but is not routine practice in all European primary care settings
- Clinical, academic, community and policy stakeholders need to work together to address implementation challenges to optimise benefits for general practitioners, patients and the broader practice population.

## Introduction

The 1978 Alma-Ata Declaration on Primary Care emphasised that people and communities have a right and responsibility to be involved in their health [1]. This policy vision is reiterated in the recent Astana Declaration from the Global Conference on Primary Healthcare [2], which promotes the involvement of individuals, families, communities and civil society through their participation in the development and implementation of policies and plans that have an impact on health. Individual and community involvement in health decision-making occurs in several ways, including to inform (i) healthcare in general practice consultations, (ii) the organisation and delivery of general practice and primary care services and (iii) the academic primary care research agenda. The first of these is usually focussed on individual involvement and is discussed in this paper in relation to *shared decision-making*. The second and third are usually focussed on community involvement and are discussed in relation *community participation* and *Public and Patient Involvement* (PPI) in research.

**Figure 1.**
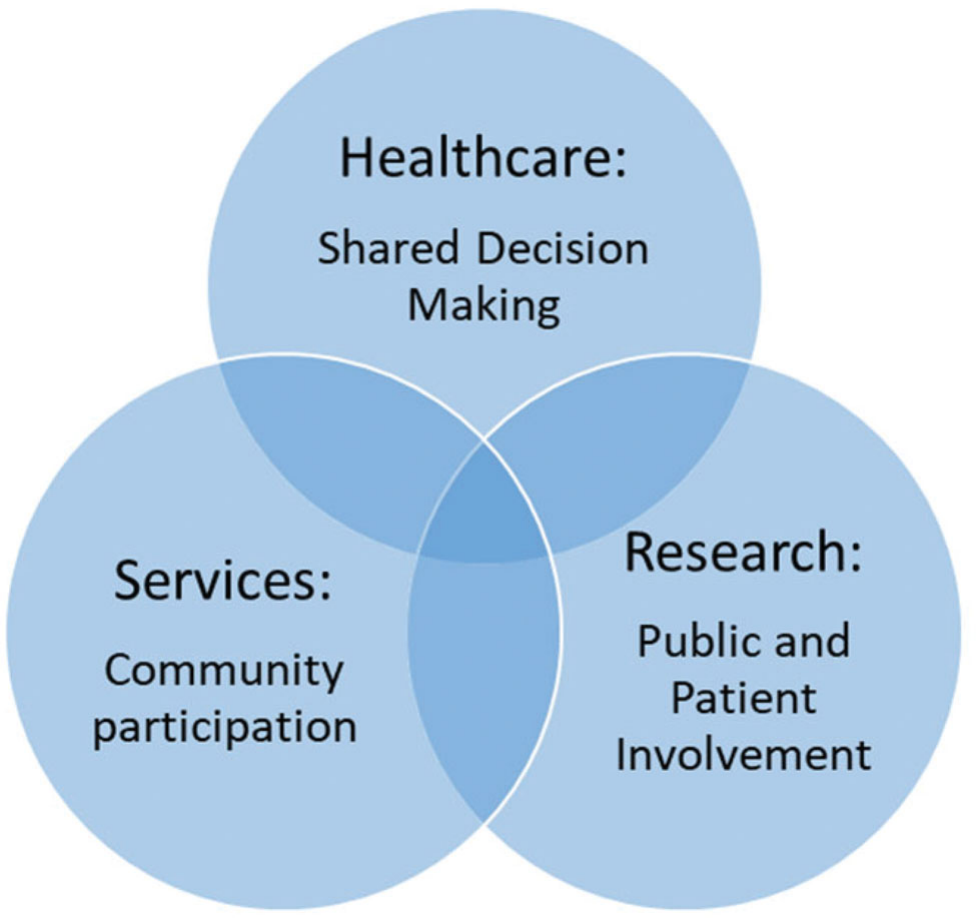
Participatory spaces for involving individuals and communities in general practice consultations and primary care settings.

Shared decision-making, community participation and PPI are represented in different literatures and are not equally familiar to general practitioners, but – and this is the key premise of this paper – thinking about them as interrelated participatory spaces in general practice and primary care is valuable (Figure 1) [3]. This is because ‘participatory spaces’ as a social science concept can be used to enhance understanding about the factors and dynamics that shape decision-making between stakeholders from different backgrounds and with different perspectives, such as doctors and patients/members of the practice population. This concept highlights that participatory spaces are influenced by physical, social and temporal issues, such as: where decisions are explored; what socio-cultural norms influence how stakeholders interact with each other; and how comfortable stakeholders are sharing decision with each other [3]. These issues provide an interesting way to analyse how participatory spaces are the same as each other and how they are different. In the next section, I will define and describe the specific features of the three participatory spaces of interest here. I will then refer to implementation challenges that are common to each of them. From there, I will consider ways to routinise individual and community participation in health decision-making in general practice consultations and primary care settings to optimise benefits for all stakeholders.

### Individual and community involvement in health decision-making. Describing spaces for participation

The idea of involving individuals in consultations is very familiar to general practitioners. It is based on a long-standing commitment in the discipline to patient-centred care [4]. This commitment is underpinned by the view that people develop expertise from their experiences of self-care practices, caring roles and interactions with healthcare professionals and the healthcare system [5]. For this reason, general practitioners are trained to be skilled communicators in order to elicit patients’ views in consultations. Shared decision-making in consultations develops and deepens the principle of patient-centred care [4]. It has been defined as: ‘an approach where clinicians and patients share the best available evidence when faced with the task of making decisions, and where patients are supported to consider options, to achieve informed preferences’ [6]. Shared decision-making occurs in consultation rooms between doctors and patients and, sometimes, carers. Research over the past 40 years has generated evidence for, and tools to promote, shared decision-making in consultations so that patients can have the experience of being involved in decision-making about their healthcare [4].

The idea of involving communities in health decision-making in the practice or community setting may or may not be very familiar to general practitioners, depending on their national policy context. WHO defines such community participation as ‘a process by which people are enabled to become actively and genuinely involved in defining the issues of concern to them, in making decisions about factors that affect their lives, in formulating and implementing policies, in planning, developing and delivering services and in taking action to achieve change’ [7]. This is a ‘bottom-up’ approach to improving health. It is underpinned by the view that people living in local communities have expertise about their broader environment and how the social determinants of health (the conditions in which they are born, live and work) shape their health [8]. This expertise ‘on the ground’ can complement the knowledge and expertise of general practitioners and other primary care professionals working in the community because it provides knowledge about community needs from the perspective of community members [9,10]. There is evidence that community participation initiatives make it more likely that communities will get the healthcare they need where and when they need it [11].

This is recognised in the discipline of general practice: efficient, robust and responsive primary healthcare relies on services knowing their local population and its needs, and fine-tuning services to provide appropriate and relevant care [8,9]. There are excellent examples of Community Oriented Primary Care (COPC) styled healthcare initiatives and COPC modelled interdisciplinary education that involve people living in the community to develop primary care services [12]. The models are based on subtle, but important, changes in terminology that encourage a shift in attention from the more biomedically focussed notion of ‘patient’ to more holistic understandings of ‘person’ and ‘people’ [9]. In some countries, there are policies that promote patient participation groups (PPGs) or community health panels to formalise the ways in which communities can ‘have a voice’ about general practice priorities, services and innovations [13]. The extent to which these structures are mandatory varies between settings: community involvement in Irish Primary Care Teams was a policy recommendation without contractual obligation [14], while PPGs were mandated in English general practice contracts in 2014 [15]. All of these models require general practitioners to have time for discussion and deliberation with people living in the community during meetings in the practice setting or in community centres to explore options, make decisions and co-design service plans.

Finally, the idea of involving communities in health research is somewhat newer and may be less familiar to general practitioners. Public and Patient Involvement in health research can be broadly defined as ‘research being carried out “with” or “by” members of the public rather than “to”, “about” or “for” them’ [16]. It is mandated or endorsed by many funding agencies including the European Commission [17]. This is underpinned by the view that patients, carers and members of the public have ideas and concerns about what research should be about, again because of their experiences and expertise about their own health experiences. This form of involvement speaks to the role of science in the development of primary care. Practice-based evidence requires general practitioners to be involved in research, as researchers. To make sure that, for example, guidelines are relevant to, and tailored for, the needs of the patient and the population at stake. The drive for PPI can be seen as an extension of that in the sense that patients and members of the public also have expertise that can guide our thinking about the development guidelines for general practice consultations [e.g. 18]. This form of community involvement in health decision-making requires a fundamental shift in perspective for general practice researchers whereby patients or members of the public are seen not only as research subjects in studies, from whom data is extracted, but also as partners and collaborators in research [19]. They can participate in steering committee meetings with academics to inform decision-making about relevant research topics, enrolling study subjects, methods for data collection, interpretation of findings and appropriate strategies for dissemination [20].

### Individual and community involvement in health decision-making: Implementation challenges and solutions

Each form of involvement described in this paper faces implementation problems. These are not necessarily routine ways of working across European general practices. SDM, despite 40 years of research and policy support, is not routine practice in general practice consultations [4]. Community participation in practice settings (via interdisciplinary teams or structures such as PPGs) does occur [21,22], but these initiatives can be difficult to sustain and are not routine across primary care. PPI in research, while gaining momentum and offering rich learning for service development, is not fully embedded as routine way of researching either [23].

Drawing on implementation theory and participatory methodologies that have proved successful for supporting European implementation projects in general practice consultations and primary care settings [24,25], there are three recommendations for a way forward that I wish to make. First, the idea of individual and community involvement in health decision-making needs to *make sense* to general practitioners so they can see its value and ‘buy into’ it. General practitioners sometimes struggle with ‘newer’ ideas and imperatives such as community participation and PPI [23,26]. Therefore, it is worth emphasising that shared decision-making, community participation and PPI are all underpinned by the same focus on patients/people as experts. Thus, while an idea sounds new, it is based on the familiar core commitment of patient-centredness. Making this connection across these different forms of involvement may help increase sense-making and ‘buy-in’ among general practitioners.

Second, involving individuals in shared decision-making and communities in health decision-making about services and research takes *time and resources*. Such interactions require discussion and deliberative thinking [4,23,25,26]. This is a serious challenge in the context of under-resourced services in the community [27]. General practitioners are trained to be pragmatic and fast thinkers because busy surgeries with (approximately) ten-minute appointment schedules rely on those skills. This status quo, however, obscures the value of other, more deliberative forms of decision-making, which are surely also inherently part of general practice as a discipline given its commitment to patient-centred care. There is evidence that when general practitioners have the training and resources to meaningfully involve individuals and communities in shared decision-making, community participation and PPI, they see the benefits and value very clearly [4,23,26].

Third, each of these participatory spaces requires *power-sharing.* Given the longstanding, traditional social hierarchies that elevate the medical profession in society [28], it is very important to know much more about the specifics of power in each one. Research by academic general practitioners, community partners and social scientists in general practice and primary care settings could explore a range of inter-related questions: How exactly does ‘doctor as expert’ meet with ‘patient as expert’ in the consultation room? What kind of adjustments are needed when doctors meet patients who are members of a PPG or a community health forum and are there as ‘people’ and ‘community members’ rather than as a patient with individual symptoms and health needs? Similarly, how do GPs identify and interact with PPI contributors if they are also their patients in the practice? These are the kind of important questions posed by general practitioner researchers at the EGPRN conference based on their initial experiences of involving patients and community in research and development projects (Vigo, October 2019), who were enthusiastic about these ideas and wanted to ‘think through’ the implications for doctor–patient relationships.

Overall, to routinise individual and community health decision-making in general practice consultations and primary care settings, there is a fundamental need to create an enabling environment for general practitioners to integrate this as part of their core work. Therefore, it is imperative that policy visions such as Astana are followed through with careful analysis of general practitioners’ roles [2]: van den Muijsenbergh and van Weel emphasise the role of international and national academic primary care organisations [29], policy makers and public health colleagues in this regard. This analysis could include a specific goal to clarify what resources are needed for general practitioners to have time for deliberative communication with patients and members of the wider practice population. In keeping with the principles of individual and community involvement, patient and community organisations should be involved in this analysis as well so their perspectives on roles and resources are taken into account.

It is also important to build capacity among general practitioners. We should continue to provide general practitioners with tools and techniques to support shared decision-making, community participation and PPI in research. Many resources are available that emphasise democratic decision-making and enhancing patient ownership of decisions [4,25,30]. Initiatives to involve general practitioners in the development and testing of such tools and techniques are important to explore how to make them accessible and useful for general practitioners to integrate into their daily work as core work.

Finally, there are important capacity building initiatives about this field among general practitioner researchers in EGPRN and the North American Primary Care Research Group (Participatory Health Research group and Patient and Clinician Engagement Program). These are precious networks because they bring like-minded general practitioners and patient/community partners together to share experiences about putting individual and community involvement in health decision-making into practice and building the evidence base about the processes and outcomes.

## Conclusion

As a community of general practice and primary care academics and clinicians, we need to reduce the barriers that prevent general practitioners from having sufficient time for involving individuals and communities in health decision-making. General practitioners can independently seek opportunities through, for example, continuing professional development or research partnerships to further develop knowledge and skills for shared decision-making, community participation and PPI in research. However, it is essential that there is also leadership in our discipline and partnership working involving all key stakeholders: policy makers, public health, international and national academic primary care organisations, patient and community organisations. Our goal should be to specify the resources and supports needed by general practitioners, to integrate the available tool and techniques for individual and community involvement into general practice routines. This will bring us closer to a situation where we can fully optimise individual and community involvement in health decision-making and harness all the benefit of working with patients/people as experts.
